# Plasmatic Levels of Cytokines and Quality of Life among Elderly Individuals with Dizziness

**DOI:** 10.1055/s-0044-1791731

**Published:** 2025-01-22

**Authors:** Gislaine da Silva Moreira, Luciana Lozza de Moraes Marchiori, Daiane Soares de Almeida Ciquinato, Glória de Moraes Marchiori, Licia Sayuri Tanaka Okamura, Braulio Henrique Magnani Branco, Regina Célia Poli-Frederico

**Affiliations:** 1 Program in Rehabilitation Sciences, Universidade Estadual de Londrina (UEL)/Universidade Pitágoras Unopar Anhanguera (UNOPAR), Londrina, PR, Brazil; 2 Interdisciplinary Laboratory of Intervention in Health Promotion (LIIPS), Universidade Cesumar (UNICESUMAR), Maringá, PR, Brazil; 3 Research Group on Physical Education, Physiotherapy, Sports, Nutrition, and Performance (GEFFEND), Universidade Cesumar (UNICESUMAR), Maringá, PR, Brazil; 4 Postgraduate Program in Health Promotion, Universidade Cesumar (UNICESUMAR), Maringá, PR, Brazil; 5 Molecular Biology Laboratory, Universidade Pitágoras Unopar Anhanguera (UNOPAR), Londrina, PR, Brazil

**Keywords:** aging, quality of life, biomarkers, dizziness

## Abstract

**Introduction** Few studies have investigated the relationship between cytokines and dizziness in elderly individuals.

**Objective** To assess the levels of inflammatory biomarkers and their relationship with quality of life (QoL) among elderly individuals with dizziness.

**Methods** We conducted a cross-sectional study with 103 participants (90 women and 13 men) who were assessed through the Visual Analogue Scale (VAS) and the Dizziness Handicap Inventory (DHI). The plasma levels of cytokines were measured through the cytometric bead array (CBA) method. Cross-tabulations with the Chi-squared test were used to verify sample homogeneity, and parametric tests were used to analyze the data.

**Results** Out of the total sample of 103 individuals, dizziness was reported by 40 women and 5 men. A difference between the groups with and without dizziness was observed regarding the levels of interleukin 4 (IL-4; *p* = 0.011): the group without dizziness presented a higher mean level (2.1 ± 2.8 pg/mL) when compared to the group that presented dizziness (1.0 ± 1.7 pg/mL). Another difference was found between the DHI classifications and the plasma levels of tumor necrosis factor alpha (TNF-α;) *p* = 0.015): the groups without any losses in QoL presented lower TNF-α levels (1.4 pg/mL) compared to the group that presented moderate QoL loss (6.3 pg/mL).

**Conclusion** Dizziness affects the functional, physical and emotional dimensions of QoL and plays a role in the decrease in the levels of IL-4 and in the presence of fullness and tinnitus. Higher VAS scores are related to dizziness in the elderly. Moreover, the increased levels of TNF-α were associated to light-to-moderate losses in QoL among elderly patients with dizziness.

## Introduction

Researchers estimate that in 2040 there will be 1 billion people in the world aged 60 years or older. With the increase in life expectancy, there is also an increase in the search for health and good quality of life (QoL) among the elderly. 1 Human aging can be considered a critical period for health, in which pathophysiological changes can cause chronic diseases, resulting in large impacts in daily activities and in health 2 maintenance, since it is a gradual process of decline with diminished capacity of survival 3 and adaptation. Aging is also called senescence, which is a common genetic process for all organisms, facilitating the onset among the elderly of chronic diseases that can diminish longevity, therefore affecting QoL. 2 3

With senescence comes immunological aging, which is associated to a progressive decline in immune function and consequent change in the production of pro- and anti-inflammatory cytokinesthe control or the negative regulation of the production of proinflammatory cytokines, such as interleukin (IL) 2 (IL-2), IL-6, tumor necrosis factor alpha (TNF-α), and interferon gamma (INF-γ). It is also important to consider the anti-inflammatory cytokine levels that neutralize inflammation, such as those of IL-4 and IL-10, which diminish with age, increasing the disability picture to maintain immunological homeostasis. 4 The golden standard method to evaluate the plasma levels of inflammatory biomarkers is flow cytometry, which will help in future biomedical interventions, including medicine or other agents that can modulate its activity. 4 5

Dizziness is a common symptom among the elderly, and it is associated with several limiting 6 7 diseases. The most prominent complaints regarding dizziness are the disturbance in the sense of position or movement, 8 which leads to physical, emotional, cognitive, and functional limitations. 9 As explained by Ribeiro et al., 9 more than 10% of the world population presents some kind of dizziness, which is more common in women and is worsened by the normal or abnormal hormonal variation, which affects the inner ear function, causing or aggravating dizziness. 10 11 The impact of dizziness on QoL has been increasingly investigated, in multifactorial and multidisciplinary 12 13 analyses, and the identification of the most affected QoL aspects can help physicians choose the best treatment or rehabilitation. 14 15

The frequency of dizziness episodes has a negative influence on QoL, 16 since dizziness may cause accidental falls and, consequently, lesions. 17 The Dizziness Handicap Inventory (DHI) 18 is a self-reported questionnaire that assesses the impact of dizziness on the physical, functional, and emotional aspects 14 of QoL. The DHI has been translated, adapted, and validated for the Brazilian population, 14 19 and it shows reliability in the initial evaluation and in the assessment of the therapeutic effect of the chosen treatment. 14

So far, few studies 42 have been conducted on the relationship between cytokines and dizziness in healthy elderly individuals. Therefore, the present study aimed to assess the levels of inflammatory biomarkers and their relationship with QoL among elderly individuals with dizziness.

## Methods

### Study Design and Sample

The present is a cross-sectional study in which all participants were informed about the procedures involved in the evaluations and signed a free and informed consent form (FICF). The study was approved by the institutional Ethics in Research Committee, under protocol number CAAE: 92480418.8.0000.5231.

The inclusion criteria were as follows: patients of both sexes aged ≥ 60 years, who were physically independent, classified as level 3 in terms of physical function, as proposed by Cordeiro 40 , with a level of oral comprehension that enabled them to fill out the DHI, who signed the FICF, filled out the questionnaires during the anamnesis stage, and had a blood sample drawn for the cytokine count. The exclusion criteria were having participated in supervised exercise programs in the previous three months, presenting the following conditions: decompensated respiratory or heart diseases; vestibular, proprioceptive or equilibrium diseases (verified by anamnesis and clinical evaluation), such as multiple sclerosis and Parkinson disease; orthopedic diseases, such as rheumatoid arthritis; cardiovascular, psychiatric, metabolic, neurological or neuropathic diseases, such as diabetes mellitus (verified by anamnesis and clinical evaluation); and cancer or recent surgeries that could interfere in the evaluation.

In order for us to perform the proposed evaluations, the sample was composed of 103 elderly individuals who were divided into 2 groups: patients with dizziness (n = 45) and patients without dizziness (n = 58).

### Evaluation Instruments

An anamnesis was performed with all participants, with questionnaires regarding demographic data, hearing, and clinical characteristics. The subjects who presented complaints of dizziness or vertigo were asked to indicate their perception of the intensity of the dizziness using the Visual Analogue Scale (VAS). The VAS is commonly used to assess pain, and it consists of a 10-cm straight line with the two endpoints labeled “no pain” and “worst pain ever”. 21 Therefore, the VAS can be used to measure the perception of dizziness symptoms from 0 to 10, with 0 corresponding to lack of dizziness and 10, to the maximum dizziness intensity felt by the patient. 22 23

Moreover the elderly subjects with dizziness complaints filled out the Brazilian version of the DHI, 14 which is composed of 25 questions on the self-perception of the impact of dizziness on 3 aspects of QoL: 7 questions evaluate the physical aspects, 9 questions evaluate the emotional aspects, and 9 questions evaluate the functional aspects.

The DHI's questions were read out loud by the interviewer, and each participant had to choose only one answer, with the options being: “yes” (4 points), “sometimes” (2 points), and “no” (0 points). The maximum score is of 100 points, and the higher the total score, the more severe the loss in QoL. A score between 0 and 25 points indicate no loss in QoL; from 26 to 50 points, light loss; from 51 to 75, moderate loss; and from 76 to 100, severe loss.

The plasma levels of cytokines of the participants were assessed through the cytometric bead array (CBA) method, using the Human Th1/Th2 Cytokine Kit II (BD Biosciences, Franklin Lakes, NJ, United States). The protocol followed was the one described by Mitelman et al.: 24 six bead populations with distinct fluorescence intensity are conjugated with a capture antibody specific for each cytokine, mixed to form the CBA and read on the FL3 channel of the flow cytometer (BD Acuri C6, BD Biosciences). The bead populations were visualized according to their respective fluorescence intensity: from least bright to the brightest. In the CBA, the cytokine capture beads are mixed with the detection antibody conjugated with the phycoerythrin (PE) fluorochrome, and afterwards, incubated with the samples.

The tubes for the acquisition were prepared with 50 μL of sample, 50 μL of the bead mixture, and 50 μL of the Th1/Th2 PE detection reagent (Human Th1/Th2 PE Detection Reagent/1 vial, 4mL; BD Biosciences). The same procedure was performed to obtain the standard curve. The tubes were homogenized and incubated for 3 hours, at room temperature, in the dark. The results were expressed as graphs and tables using cytokine levels in quantitative formats and were generated by the FCAP Array software (Soft Flow Ltd., Pécs, Hungary), version 3. The detection limits were as follows: CBA Th1/Th2: IL-2 (2.6 pg/mL), IL-4 (2.6 pg/mL), IL-10 (2.8 pg/mL), TNF-α (2.8 pg/mL), and INF-γ (7.1 pg/mL).

### Statistical Analysis

The data obtained was analyzed using the IBM SPSS Statistics for Windows (IBM Corp., Armonk, NY, United States) software, version 21.0. An association analysis of the categorical variables was performed using the Chi-squared (χ ^2^ ) test. The homogeneity of the continuous data was verified through the Shapiro-Wilk test. The associations involving the continuous and parametric variables were assessed using the student *t* -test, and analysis of variance (ANOVA) was used to find differences between the average of the crossed categorical variables and the continuous numeric variables. For all the analyzed data, the significance level adopted was *p* < 0.05 with a reliability interval of 95%.

## Results

Out of the total sample of 103 individuals, dizziness was reported by 40 women and 5 men ( Table 1 ). No statistically significant statistical differences between the groups with and without dizziness were observed regarding the following variables: sex, diabetes, hypertension, thyroid, cholesterol, triglycerides, and hearing loss; statistically significant differences were only observed for the aural fullness and tinnitus variables: 46 participants without dizziness did not present aural fullness, and 22 subjects with dizziness did; moreover, 29 participants with dizziness presented tinnitus.

**Table 1:** Categorical data of the study sample

Variables		Without dizziness: n (%)	With dizziness: n (%)	*p* -value (Chi-squared)
**Sex**	Female	50 (86.2)	40 (88.9)	0.684
	Male	8 (13.8)	5 (11.1)	
**Aural fullness**	No	46 (86.8)	23 (51.1)	0.001*
	Yes	7 (13.2)	22 (48.9)	
**Tinnitus**	No	31 (59.6)	16 (35.6)	0.018*
	Yes	21 (40.4)	29 (64.4)	
**Diabetes**	No	45 (77.6)	30 (69.8)	0.374
	Yes	13 (22.4)	13 (30.2)	
**Hypertension**	No	22 (42.3)	19 (44.2)	0.854
	Yes	30 (57.7)	24 (55.8)	
**Thyroid problems**	No	40 (76.9)	31 (72.1)	0.590
	Yes	12 (13.1)	12 (13.1)	
**High Cholesterol**	No	28 (53.8)	22 (51.2)	0.794
	Yes	24 (46.2)	21 (48.8)	
**Triglycerides**	No	36 (69.2)	32 (74.4)	0.577
	Yes	16 (30.8)	11 (25.6)	
**Hearing loss**	No	12 (20.7)	4 (8.9)	0.101
	Yes	46 (79.3)	41 (91.1)	

**Note:** *Statistically significant.

Regarding the inflammatory markers, a statistically significant difference was observed between both study groups for the levels of IL-4 ( *p* = 0.011): the group without dizziness presented a higher mean level (2.1 ± 2.8 pg/mL) when compared to the group with dizziness (1.0 ± 1.7 pg/mL), as indicated in Table 2 . No statistically significant differences were observed for the other cytokines ( *p* > 0.05). The mean dizziness score on the VAS and the mean scores on each domain of the DHI, as well as the mean total DHI score, were significantly higher in the group with dizziness compared to the group without dizziness ( *p* < 0.05).

**Table 2:** Plasma levels of inflammatory markers among the study sample

Variables	Without dizziness (n = 58): mean ± standard deviation	With dizziness (n = 45) : mean ± standard deviation	*p* -value (independent *t* -test)
**Age (years)**	71.7 ± 7.8	69.4 ± 7.1	0.121
**INF-γ (pg/mL)**	10.4 ± 15.2	7.4 ± 10.2	0.259
**TNF-α (pg/mL)**	3.9 ± 5.3	2.8 ± 3.9	0.235
**IL-10 (pg/mL)**	2.3 ± 2.6	3.2 ± 10.8	0.511
**IL-6 (pg/mL)**	2.7 ± 3.2	3.5 ± 7.7	0.448
**IL-4 (pg/mL)**	2.1 ± 2.8	1.0 ± 1.7	0.011*
**IL-2 (pg/mL)**	3.2 ± 4.3	2.9 ± 4.3	0.818
**VAS -** **dizziness**	1.2 ± 1.8	5.2 ± 2.6	0.001*
**DHI - functional**	2.3 ± 3.9	10.6 ± 9.6	0.001
**DHI - emotional**	3.2 ± 5.7	9.8 ± 9.8	0.001*
**DHI - physical**	2.6 ± 4.4	11.7 ± 7.9	0.001*
**DHI - total**	8.2 ± 11.3	32.0 ± 24.7	0.001*

**Abbreviations:** DHI, Dizziness Handicap Inventory; IL, interleukin; INF-γ, interferon gamma; TNF-α, tumor necrosis factor alpha; VAS, Visual Analogue Scale.

**Note:** *Statistically significant.

Differences were found between the DHI classification and the plasma levels of TNF-α ( *p* = 0.015): the subjects who did not present QoL loss presented lower mean levels of TNF-α (1.4 ± 2.6 pg/mL) compared to the subjects with moderate QoL loss (6.3 ± 5.9 pg/mL), as shown in Table 3 .

**Table 3:** Comparative analysis regarding the DHI classification, age and inflammatory markers (n = 40)

Variables	Without QoL loss (19)	Light QoL loss (10)	Moderate QoL loss (9)	Severe QoL loss (2)	*p* -value (ANOVA)
**Age (years)**	69.2 ± 6.8 ^a^	67.7 ± 5.6	71.9 ± 6.6	64.0 ± 1.4	0.337
**INF-γ (pg/mL)**	5.8 ± 9.0	6.6 ± 8.9	10.2 ± 12.8	24.2 ± 13.0	0.106
**TNF-α (pg/mL)**	1.4 ± 2.6	3.0 ± 2.8	6.3 ± 5.9	0.0 ± 0.0	0.015*
**IL-10 (pg/mL)**	1.5 ± 2.0	1.1 ± 2.2	9.7 ± 23.4	2.8 ± 4.0	0.217
**IL-6 (pg/mL)**	2.3 ± 2.6	7.1 ± 15.7	3.0 ± 2.7	1.4 ± 1.9	0.500
**IL-4 (pg/mL)**	0.7 ± 1.7	0.9 ± 1.5	1.9 ± 2.3	2.4 ± 3.3	0.316
**IL-2 (pg/mL)**	2.6 ± 4.2	2.9 ± 4.2	2.6 ± 3.4	0.0 ± 0.0	0.214

**Abbreviations:** ANOVA, analysis of variance; DHI, Dizziness Handicap Inventory; IL, interleukin; INF-γ, interferon gamma; QoL, quality of life; TNF-α, tumor necrosis factor alpha.

**Notes:** The values on the table refer to mean ± standard deviation; *statistically significant.

## Discussion

With the goal of assessing the effect of inflammatory biomarkers in the QoL of elderly individuals with dizziness according to the DHI, in the present study, we observed a difference in the levels of the anti-inflammatory cytokine IL-4 between the groups: those without dizziness presented increased levels (2.1 ± 2.8 pg/mL) compared to those with dizziness (1.0 ± 1.7 pg/mL). Increased levels of IL-4 suggest a protective role against dizziness in these elderly patients, because the role of IL-4 is to neutralize inflammation. In line with these findings, Furukawa et al. 25 reported an anti-inflammatory effect of IL-4 on inflammatory lesions in the inner ears of guinea pigs with autoimmune diseases, using transplanted stem cells that expressed IL-4.

The associations found between dizziness and tinnitus and between dizziness and aural fullness are well described in the literature. 26 Studies have also shown that dizziness and hearing loss are risk factor for tinnitus, 27 28 making such individuals prone to feel more depressed, with diminished QoL and psychological well-being. 29 30 The data was verified in this study which presented dizziness and tinnitus when having hearing loss and diminished QL.

The association found in the present study between the plasma levels of pro-inflammatory cytokine TNF-α in the group with dizziness with moderate QoL loss according to the DHI indicates a possible contribution of TNF-α in increasing the discomfort caused by dizziness, which impacts QoL. Fujioka et al 20 observed that an increase in TNF-α expression worsened cochlear function, as a self-protection mechanism, inducing proinflammatory cytokine production. However, Gazquez et al. 31 investigated TNF-α expression in Ménière disease (MD), where the association of the clinical aspects were tested as vertigo with the disease progression, and the conclusion was that it is not possible to associated functional variables of pro-inflammatory cytokines (including TNF-α) with susceptibility and progression of hearing loss.

The results of the present study indicate an association involving the dizziness intensity according to the VAS and the functional and emotional DHI subscales, making the perception of dizziness more intense, which causes more impairment in the QoL. The physical subscale of the DHI assesses the onset or worsening of dizziness when certain body movements are performed, whereas the functional and emotional subscales measure the impact of dizziness 32 on daily-life and social activities.

The physical impairment caused by chronic dizziness and by body imbalance provokes less objective issues such as insecurity, irritability, fear of going out alone, fear that this is a symptom of an untreatable disease, the feeling of being disconnected from reality, anxiety, 33 34 35 36 depression, and panic. 35 36 Besides the physical and emotional distress, patients with dizziness may present worse performance in functionality in daily-life activities and in professional and/or social 28 activities. Thus, Dizziness has a negative 35 37 impact on QoL.

In a randomized controlled trial on the efficacy of vestibular rehabilitation, Tokle et al. 38 pointed out that the DHI presents good sensitivity in the evaluation of physical, functional and emotional aspects, because they play an important role in the clinical condition of dizziness. Another study 39 using questionnaires to evaluate patients before and after an intervention compared three exercise programs for the treatment of patients complaining about persistent dizziness after a neck injury. Therefore, proper evaluations and interventions regarding the physical, emotional and functional issues resulting from dizziness are necessary to optimize and improve QoL for these patients.

It should be noted that there are few studies on the effect of inflammatory biomarkers in the QoL of elderly individuals with dizziness; however, some studies have provided data that can serve as a basis for future treatments related to otoneurological changes such as dizziness, vertigo, tinnitus, and hearing loss. A study 40 on the associations involving the auditory handicap found in the Hearing Handicap Inventory for the Elderly-Screening Version (HHIE-S) questionnaire and hearing loss and the plasma levels of inflammatory biomarkers of 76 participants, used the flow cytometry method to measure the plasma levels of IFN-γ; an inverse correlation was observed between the increase in the plasma levels of IFN-γ and the normal auditory handicap ( *p* = 0.015; rs = -0.280). The authors 40 also observed that, the higher the levels of IFN-γ, the lower the restrictions of the elderly in terms of daily-life activities, family relationships, and social interactions.

Another study 41 demonstrated that the pathological mechanism leading to sporadic MD is still poorly understood; however, an allergic inflammatory response seems to be involved in some patients with MD. The study 41 performed mass cytometry immune to was objective decipher an immune signature associated with the syndrome, identified two clusters of individuals according to the single cell cytokine profile. These clusters presented differences in immunoglobulin E (IgE) levels, immune cell population abundance, including a reduction in CD56dim NK-cells, and changes in cytokine expression with a different response to bacterial and fungal antigens. The results support a systemic inflammatory response in some MD patients who show a type-2 response with allergic phenotype, who could benefit from personalized IL-4 blockers. 41

The present study has a few limitations, such as the small sample size; for example, only 2 patients in the sample presented severe QoL loss according to the DHI, which can be insufficient for a subgroup analysis of secondary results. Other limitations are related to the clinical collection nature of subjective data, since this condition was reduced by the utilization of biological markers for the interleukins significantly associated to the results. Moreover, another limitation is the fact that we did not specifically recruit elderly individuals based on dizziness complaints. Nonetheless, the sample was composed of a homogeneous group of individuals, which makes the results reliable.

Considering the lack of research associating dizziness to the increase in the levels of inflammatory biomarkers in the elderly, future studies could consider recruiting only patients with dizziness complaints, to best explore this symptom and provide more accurate results. Just as, distribution identification of inflammatory markers can help professionals from multidisciplinary health teams, when attending to the elderly, to track illnesses or adverse events and monitor the effects or the effectiveness of therapeutic responses. This way, it becomes possible to recognize the vulnerable groups for an early rehabilitation intervention.

## Conclusion

Dizziness affects the functional, physical, and emotional aspects of QoL among the elderly, and it decreases the levels of anti-inflammatory cytokine IL-4. The presence of aural fullness and tinnitus, as well as higher scores on the VAS, are related to dizziness in the elderly. Moreover, we verified that higher levels of proinflammatory cytokine TNF-α were associated to light-to-moderate QoL loss among elderly patients presenting dizziness.
